# From Body Mass Index to Biology: Reconciling Diagnostic Clarity and Surgical Eligibility in Obesity Care

**DOI:** 10.1007/s11695-025-08411-5

**Published:** 2025-12-10

**Authors:** Mohamed Hany, Mohamed H. Zidan, Ahmed El Shamarka, Ricardo V. Cohen

**Affiliations:** 1 Department of Surgery, Alexandria Medical Research Institute, Alexandria University, Alexandria, Egypt; 2 The Center of Excellence in Bariatric and Metabolic Surgery, Madina Women’s Hospital, Alexandria, Egypt; 3 The Research Papyrus Lab, Alexandria, Egypt; 4 Alexandria University, Alexandria, Egypt; 5 The Center for Obesity and Diabetes, Hospital Alemão Oswaldo Cruz, São Paulo, Brazil; 6 International Federation of Obesity Surgery and Other Therapeutics (IFSO) Global, Napoli, Italy

For over thirty years, the eligibility criteria for metabolic-bariatric surgery (MBS) have rested mainly on a body mass index (BMI)-centric thresholds established by the 1991 North American National Institute of Health (NIH) guidelines [1]. While this framework is straightforward operationally, it has faced substantial criticism for inadequately representing the biological and functional diversity among individuals with obesity [2, 3].

Two important documents have recently emerged to address these shortcomings, each from a distinct vantage point [4, 5]. The 2022 joint guidelines by the American Society of Metabolic and Bariatric Surgery (ASMBS) and the International Federation for the Surgery and Other Therapies for Obesity (IFSO) advocate for a more inclusive model for surgical eligibility [4, 6]. The guidelines propose lowering thresholds to include individuals with a BMI ≥ 35 kg/m², regardless of comorbidities, and those with a BMI of 30–34.9 kg/m² who have metabolic diseases [4, 6]. This shift reflects growing evidence of the efficacy and safety of MBS across a wider demographic and emphasizes access and metabolic risk mitigation [6].

Notably, these eligibility updates, including patients with BMI ≥ 35 kg/m² without comorbidities, were adopted based on expert consensus, underscoring the urgency to expand treatment while evidence continues to evolve [6]. Table 1 shows the differences between the 1991 NIH consensus and the 2022 ASMBS/IFSO guidelines.

**Table 1:** This table compares the original 1991 NIH eligibility criteria with the updated 2022 ASMBS/IFSO guidelines, highlighting the transition from strictly weight- and comorbidity-based thresholds to a more inclusive and risk-oriented model. The updated criteria broaden access to metabolic-bariatric surgery by removing mandatory comorbidity requirements at BMI ≥ 35 kg/m², introducing lower BMI thresholds for high-risk ethnic groups, and emphasizing earlier metabolic intervention

Domain	NIH 1991 Consensus	ASMBS/IFSO 2022 Guidelines
Primary Eligibility Criterion	BMI ≥ 40 kg/m² or ≥ 35 kg/m² with comorbidities	BMI ≥ 35 kg/m² regardless of comorbidities; 30–34.9 kg/m² with metabolic disease
Comorbidity Requirement	Required for BMI 35–39.9 kg/m²	Not required at BMI ≥ 35 kg/m²
Age Recommendation	18–60 years	No strict age limits; individualized assessment
Ethnic-Specific Adjustments	Not addressed	Lower BMI thresholds for high-risk ethnic groups (e.g., ≥ 27.5 kg/m² in Asians)
Preventive Focus	Tertiary (post-complication)	Earlier metabolic intervention encouraged
Surgical Indication	Severe obesity with comorbidities	Recognized as a treatment for metabolic disease, even in lower BMI groups

Metabolic disease refers to conditions such as type 2 diabetes, dyslipidemia, or hypertension attributable to adipose dysfunction, consistent with ASMBS/IFSO definitions

In contrast, the 2025 Lancet Diabetes & Endocrinology Commission provides a paradigm shift in diagnosing obesity [5]. It moves beyond anthropometric measures to a functional perspective, differentiating between preclinical obesity, characterized by excess adiposity without organ dysfunction, and clinical obesity, where there is evidence of tissue damage or functional impairment [5]. As with all chronic diseases, this definition aims for a pathophysiology-accurate model that enhances diagnosis, staging, and patient outcomes. Table 2 summarizes the Lancet Commission’s definitions of pre-clinical and clinical obesity.

**Table 2:** Functional staging of obesity according to the 2025 lancet commission. obesity is defined in individuals with a BMI ≥ 30 kg/m² plus at least one additional anthropometric indicator of excess adiposity (increased waist circumference or waist-to-hip ratio, or waist-to-height ratio). Alternatively, in the absence of a BMI ≥ 30, the presence of at least two abnormal anthropometric markers May also define the individual with obesity. When available, direct measures of body composition (e.g., DEXA) can provide a more accurate assessment of excess or dysfunctional adiposity. After this confirmation of excess adiposity, the commission classifies obesity into preclinical and clinical stages based on the presence of functional impairment and organ/system dysfunction attributable to excess adiposity

Category	Preclinical Obesity	Clinical Obesity
Definition	Excess adiposity without measurable organ dysfunction or significant functional impairment	Excess adiposity with demonstrable organ/system dysfunction or substantial limitation in daily functioning
Diagnostic Criteria	Presence of elevated body fat measured through anthropometric parameters without signs/symptoms attributable to adiposity	Evidence of metabolic, mechanical, or impairment of daily life activities with a pathophysiological link with excess adiposity.
Functional Status	Normal or near-normal physiological and functional capacity	Reduced physiological reserve, functional limitations, or evidence of obesity-induced disease
Recommended Approach	Lifestyle optimization, monitoring, and risk prevention; medical and surgical interventions may be considered in special cases where rapid risk reduction is necessary to facilitate or expedite other treatments like organ transplantation, cancer therapy, or major orthopedic surgery, and the treatment of type 2 diabetes.	Active intervention recommended (e.g., pharmacotherapy, metabolic surgery); management tailored to disease severity and impact on quality of life

“Functional impairment” refers to limitations in activities of daily living or physiological reserve. “Organ/system dysfunction” refers to measurable metabolic, mechanical, or psychological abnormalities directly attributable to adipose excess (e.g., insulin resistance, OSA, NAFLD)

This editorial aims to clarify how the treatment-oriented ASMBS/IFSO eligibility criteria and the diagnostic framework proposed by the 2025 Lancet Commission can be understood as complementary rather than conflicting, and how their integration supports a proportional, risk-stratified approach to contemporary obesity care.

However, it is crucial to recognize that these guidelines and the 2025 Lancet Diabetes & Endocrinology Commission framework serve fundamentally different roles. The ASMBS/IFSO guidelines are treatment-oriented, establishing criteria for those who may benefit from MBS [4, 6]. In contrast, the 2025 Lancet Commission proposes a diagnostic framework that defines obesity based on functional impairment and pathophysiology (Fig. 1) [5]. Rather than conflicting, these frameworks are complementary: one addresses therapeutic eligibility while the other refines disease definition. Integrating both can enable more precise and ethically sound clinical decisions. Within this functional paradigm, the Lancet Commission’s model serves as the conceptual anchor for proportional, staged decision-making, and the subsequent discussion builds on this framework without re-articulating its definitional elements [5].

**Fig. 1:**
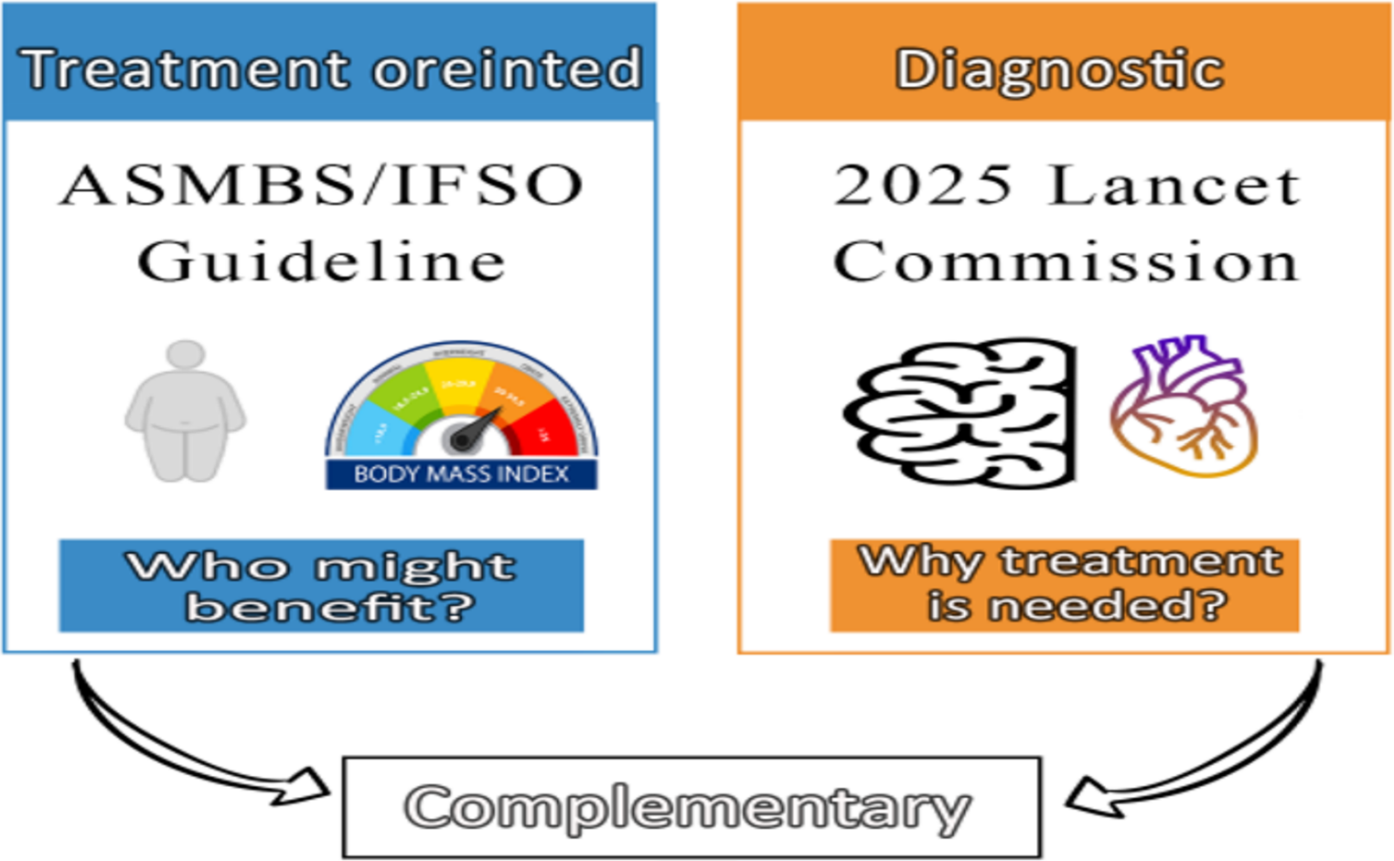
Conceptual relationship between the treatment-oriented ASMBS/IFSO metabolic-bariatric surgery eligibility guidelines and the diagnostic framework proposed by the 2025 Lancet Diabetes & Endocrinology Commission. The ASMBS/IFSO guideline (left panel) focuses on who can be treated and who might benefit from surgery, whereas the Lancet Commission (right panel) explains why treatment is needed by defining obesity as a clinical disease when excess adiposity causes organ/system dysfunction and functional impairment. The bidirectional arrows and the label “Complementary” highlight that these frameworks are designed to work together rather than compete, aligning surgical eligibility with disease biology and treatment urgency

Importantly, the Lancet Commission does not seek to restrict access to therapy, but rather to ensure that surgical or pharmacologic treatment is proportionate to disease severity [5]. In preclinical obesity, the Commission advocates for longitudinal follow-up, with timely escalation when risk indicators emerge or worsen [5]. This approach aligns with principles used in other chronic conditions, where early risk is monitored rather than treated invasively.

An apt analogy lies in colorectal cancer prevention, where the presence of adenomatous polyps prompts surveillance and preventive strategies, not immediate colectomy, despite the potential risk of evolving into malignancy [7]. Similarly, preclinical obesity signals biological vulnerability, warranting active monitoring but not necessarily immediate intervention.

Thus, rather than introducing gaps, the Commission reframes obesity management through a logic of proportionality. The apparent tension is not between therapeutic inclusion and diagnostic exclusion, but between universal access and calibrated urgency. Addressing this will require developing tools that account not only for current dysfunction but also for trajectory, context, and risk potential, ensuring that treatment is equitable and timely. Taken together, these considerations set the stage for a unified understanding of how diagnostic and therapeutic frameworks can operate synergistically in clinical practice.

## Complementary Frameworks for Treatment and Diagnosis

The ASMBS/IFSO guidelines serve primarily as a therapeutic framework defining who can access metabolic surgery, whereas the Lancet Commission provides a diagnostic framework clarifying who has biologically measurable disease and how severe it is. In parallel, complementary frameworks for treatment and diagnosis emerge when their distinct purposes, one diagnostic and one therapeutic, are considered together. Having established their distinct purposes, one diagnostic, the other therapeutic, it becomes clear that the ASMBS/IFSO guidelines and the 2025 Lancet Diabetes & Endocrinology Commission framework are not contradictory, but instead offer complementary strengths within a modern, risk-stratified approach to obesity care.

The ASMBS/IFSO guidelines broaden access to MBS by recognizing the limitations of BMI as a solitary criterion and acknowledging that metabolic dysfunction can exist below traditional thresholds [4]. Their pragmatic orientation promotes earlier intervention to prevent irreversible complications, particularly in populations historically underserved by rigid BMI-based policies. A key limitation, however, is the scarcity of high-quality evidence supporting surgery for patients with a BMI >35 kg/m² in the absence of comorbidities or functional impairment. Much of the support for this population stems from subgroup analyses and observational studies not explicitly designed for this indication [6], raising concerns about potential overtreatment if clinical staging is not adequately incorporated.

In parallel, the Lancet Commission framework introduces a diagnostic model rooted in pathophysiology and functional impairment, distinguishing between preclinical and clinical obesity based on organ/systemic dysfunction rather than BMI alone [5]. This model supports proportional treatment allocation, mirroring chronic disease frameworks used in oncology and nephrology. Importantly, it does not advocate withholding care arbitrarily; instead, it allows for early surgical or pharmacologic intervention when rapid risk mitigation is clinically warranted, such as in pre-transplant patients or those with accelerating metabolic decline [5].

Together, these frameworks offer distinct but synergistic contributions:

- The Lancet Commission offers diagnostic clarity and an ethical basis for staging disease severity and determining treatment urgency.
- The ASMBS/IFSO guidelines supply the therapeutic infrastructure and operational criteria to implement interventions, especially in contexts where access has historically been limited.

Nonetheless, clinical uncertainty may arise in cases where a patient meets BMI-based surgical eligibility criteria but does not yet demonstrate functional impairment under the diagnostic model. Addressing this will require objective, scalable tools integrating biological markers of adipose dysfunction (e.g., Visceral Adiposity Index) [8] and assessments of real-world feasibility for conservative management. These tools could help operationalize the Commission’s logic within the broader access framework of ASMBS/IFSO.

The ASMBS/IFSO guidelines define who can be treated, while the Lancet Commission framework clarifies who should be prioritized and when. Their thoughtful integration, grounded in proportionality, risk stratification, and clinical realism, has the potential to support a more ethically coherent, patient-centered model for obesity management.

Taken together, these frameworks can be operationalized jointly in routine practice: BMI-based eligibility allows timely access for individuals who may not yet show overt dysfunction, while functional staging helps determine treatment urgency and proportionality. For example, patients with BMI ≥ 35 kg/m² but preserved organ function may still benefit from early intervention to prevent progression, whereas preclinical patients with accelerating metabolic risk, transplant requirements, or severe psychosocial burden may appropriately receive earlier escalation despite not yet meeting clinical criteria. This integrated approach supports consistent, ethically aligned decision-making across diverse clinical scenarios.

## Integrating Diagnostic Staging with Treatment Eligibility in Obesity Care

As obesity care evolves, a recurring challenge is the potential mismatch between surgical eligibility based on BMI thresholds and diagnostic criteria rooted in functional impairment. For instance, patients with a BMI ≥ 35 kg/m² may qualify for metabolic surgery under the 2022 ASMBS/IFSO guidelines [4], yet do not meet criteria for clinical obesity under the 2025 Lancet Commission framework if they lack evidence of organ dysfunction or systemic impact [5].

This divergence is not a contradiction but a reflection of two different, valid goals: ensuring access versus ensuring proportionality. While broad BMI-based criteria aim to expand treatment availability and preempt disease progression, they risk overtreatment in individuals without current biological harm. Conversely, delaying intervention until overt dysfunction emerges may miss opportunities for early, risk-reducing action, especially in the presence of known drivers like visceral adiposity, inflammatory activity, or neuroendocrine disruption [9].

Crucially, the Lancet Commission framework does not preclude early intervention. It supports escalation when rapid risk reduction is clinically justified, including in contexts such as transplant eligibility, cancer treatment planning, or severe psychosocial distress [5]. However, clinicians require tools that translate diagnostic nuance into actionable decisions to apply this model effectively.

Therefore, the key need is for integrated stratification strategies beyond binary models. This includes combining biological markers (e.g., the Visceral Adiposity Index), behavioral and contextual assessments, and trajectory-based indicators to identify patients at high risk of progression or treatment failure, even without established comorbidities.

By moving toward a dynamic, precision-based model that blends eligibility with diagnostic rigor, clinicians can better determine who qualifies and who most needs treatment, when, and why. This shift enables more targeted, ethical, and effective obesity care, grounded in both access and appropriateness.

## Limitations of Existing Obesity Staging Systems

While the Lancet Commission framework offers a pathophysiological basis for diagnosing obesity, existing staging systems remain limited in their ability to operationalize this approach [5]. A central example is the Edmonton Obesity Staging System (EOSS) [10], which, despite its clinical utility, has three limitations directly relevant to this framework [11]. First, EOSS is inherently reactive: it infers severity only after metabolic, mechanical, or psychological complications have occurred, making it unsuitable for identifying patients in the preclinical phase [5].

One of the most widely used staging systems, the Edmonton Obesity Staging System (EOSS), has brought clinical value by incorporating medical, mental, and functional consequences of obesity [10]. However, it fundamentally assesses the outcomes of the disease rather than the disease process itself [11]. By relying on complications to infer severity, EOSS may delay staging until after significant harm, diverging from the Lancet Commission’s aim to intervene earlier based on biological dysfunction, such as adipose inflammation or neuroendocrine disruption [5]. Second, EOSS lacks integration of mechanistic or biological markers such as adipose dysfunction, inflammation, or neuroendocrine disruption, which are foundational to the Commission’s definition of clinical obesity. Third, EOSS does not incorporate contextual feasibility, including mobility barriers, caregiving demands, socioeconomic strain, or psychiatric comorbidity, all of which determine whether lifestyle-based therapy is realistically achievable [12].

In this sense, EOSS fails to align with the Lancet Commission’s call for personalized, proportional care that matches treatment intensity to both biological burden and real-world feasibility, overlooking early disease progression, contextual risk, and behavioral capacity. It is inherently limited in its ability to guide timely, individualized intervention, particularly in patients occupying the “grey zone” between BMI-based eligibility and functional symptomatology.

Therefore, while EOSS remains a helpful tool for estimating comorbidity burden, it does not fulfill the diagnostic, prognostic, or practical requirements needed to implement the Lancet Commission’s model. Obesity care will require the development of new stratification tools that integrate biological markers, disease trajectory, and contextual feasibility. Such tools would allow clinicians to identify patients at earlier stages of dysfunction and to tailor intervention intensity accordingly, mainly before irreversible complications arise. In parallel to biological staging, operationalizing diagnostic principles requires tools that translate disease mechanisms into actionable decisions.

## Translating Diagnosis into Action: Integrating Biological Risk and Lifestyle Feasibility

As obesity care moves towards precision-based stratification, future frameworks must integrate biological, functional, and real-world capacities for non-surgical treatments. This is especially important for patients eligible based on BMI but lacking functional impairment, or those who are overweight with early metabolic disturbances.

The surgical community has increasingly unraveled the biological mechanisms behind treatment resistance, such as neuroendocrine disruption and altered energy homeostasis [13]. However, we have yet to match these insights with equally sophisticated tools that assess the feasibility and appropriateness of conservative care in individual patients. Torensma et al. emphasize the need for early escalation in such physiological resistance, reinforcing the imperative for risk-adapted decision-making [13]. Nevertheless, lifestyle capability remains underexamined as a driver of clinical outcomes, and staging models often fail to account for contextual barriers that shape real-world adherence.

Despite wide variability in success, lifestyle modification is often prescribed as the first-line approach, particularly in preclinical cases [14]. Factors such as musculoskeletal limitations, inflexible work demands, caregiving responsibilities, psychiatric conditions, and socioeconomic adversity can make sustained behavioral change unfeasible, even in patients without overt comorbidities [15–17]. However, most frameworks do not incorporate these contextual constraints, leaving clinicians without objective guidance on when to escalate therapy based on feasibility.

To address this, we propose the development of validated lifestyle capability metrics, tools designed to quantify not intent or motivation, but structural, psychosocial, and physical ability for behavioral adherence. We explicitly note that these metrics represent a conceptual proposal and will require rigorous methodological development and validation before they can be applied in routine clinical decision-making. Such metrics could help reclassify patients unlikely to succeed with conservative approaches, prompting timely intervention before complications arise. Conversely, they may identify patients for whom non-invasive strategies remain viable, supporting more proportionate and individualized care.

Biologically anchored scores like the Visceral Adiposity Index (VAI) can assess metabolic dysfunction in cases where BMI and clinical presentation don’t align [8]. VAI is an example of a risk indicator, not a comprehensive staging system. As a validated measure of visceral fat and cardiometabolic risk, VAI can help identify individuals at high risk of disease progression, particularly in the preclinical phase [8, 18, 19].

However, it is critical to recognize VAI’s limited scope. While it captures important aspects of metabolic risk, it does not encompass the full spectrum of obesity-related dysfunction emphasized by the Lancet Commission [5]. Patients with substantial disease burden manifesting outside the metabolic axis, such as severe osteoarthritis, sleep-disordered breathing, or disabling psychological distress, may be misclassified if risk stratification is based solely on metabolic indices. In this light, VAI should be seen as a complementary, not a standalone, marker, useful for quantifying one dimension of risk but insufficient as a global staging tool. For this reason, VAI should be viewed as a complementary marker that quantifies only one dimension of risk.

Perhaps the most valuable contribution of these tools lies in their potential to bridge the gap between disease definition and clinical action. The Commission offers a diagnostic lens rooted in systemic dysfunction. Tools such as VAI and future lifestyle capability metrics provide operational mechanisms for translating that diagnostic lens into timely, proportional care. Recognizing this distinction not only advances precision in clinical pathways but also reinforces a key ethical imperative: that treatment intensity should be proportionate to both the disease burden and the realistic likelihood of success.

Ultimately, reconciling treatment eligibility with diagnostic clarity requires more than conceptual alignment. It calls for practical tools that quantify biological dysfunction and treatment feasibility in real-world contexts. As obesity care advances toward greater precision and personalization, the next critical step is not merely redefining the disease, but empowering clinicians to translate that definition into proportionate, ethical, and effective care.

## Data Availability

No datasets were generated or analysed during the current study.

## References

[1] NIH conference. Gastrointestinal surgery for severe obesity. Consensus Development Conference Panel. Ann Intern Med 1991, 115(12):956–961. 1952493

[2] Cohen RV, Prager G, Salminen P. Obesity is more than a number: a framework for treatment. Br J Surg. 2025. 10.1093/bjs/znaf100. 40314073 10.1093/bjs/znaf100

[3] The Lancet D. amp, Endocrinology. Redefining obesity: advancing care for better lives. Lancet Diabetes Endocrinol. 2025;13(2):75. 10.1016/S2213-8587(25)00004-X. 39826562 10.1016/S2213-8587(25)00004-X

[4] Eisenberg D, Shikora SA, Aarts E, Aminian A, Angrisani L, Cohen RV, et al. 2022 American Society of Metabolic and Bariatric Surgery (ASMBS) and International Federation for the Surgery of Obesity and Metabolic Disorders (IFSO) Indications for Metabolic and Bariatric Surgery. Obes Surg. 2023;33(1):3–14. 10.1007/s11695-022-06332-1. 36336720 10.1007/s11695-022-06332-1 PMC9834364

[5] Rubino F, Cummings DE, Eckel RH, Cohen RV, Wilding JPH, Brown WA, et al. Definition and diagnostic criteria of clinical obesity. Lancet Diabetes Endocrinol. 2025;13(3):221–62. 10.1016/s2213-8587(24)00316-4. 39824205 10.1016/S2213-8587(24)00316-4 PMC11870235

[6] De Luca M, Shikora S, Eisenberg D, Angrisani L, Parmar C, Alqahtani A, et al. Scientific evidence for the updated guidelines on indications for metabolic and bariatric surgery (IFSO/ASMBS). Surg Obes Relat Dis. 2024. 10.1016/j.soard.2024.05.009. 39419572 10.1016/j.soard.2024.05.009

[7] Screening PDQ, Prevention Editorial B. Colorectal Cancer Prevention (PDQ^®^): Health Professional Version. PDQ cancer information summaries. edn. Bethesda (MD): National Cancer Institute (US); 2002.

[8] Amato MC, Giordano C, Galia M, Criscimanna A, Vitabile S, Midiri M, Galluzzo A. Visceral adiposity index: a reliable indicator of visceral fat function associated with cardiometabolic risk. Diabetes Care. 2010;33(4):920–2. 10.2337/dc09-1825. 20067971 10.2337/dc09-1825 PMC2845052

[9] Amato MC, Giordano C. Visceral adiposity index: an indicator of adipose tissue dysfunction. Int J Endocrinol. 2014;2014:730827. 10.1155/2014/730827. 24829577 10.1155/2014/730827 PMC4009335

[10] Sharma AM, Kushner RF. A proposed clinical staging system for obesity. Int J Obes. 2009;33(3):289–95. 10.1038/ijo.2009.2. 10.1038/ijo.2009.2 19188927

[11] Callahan EA. Translating knowledge of foundational drivers of obesity into practice: Proceedings of a workshop series. 2023. 37639520

[12] Lavie CJ, Laddu D, Arena R, Ortega FB, Alpert MA, Kushner RF. Healthy weight and obesity prevention: JACC health promotion series. J Am Coll Cardiol. 2018;72(13):1506–31. 10.1016/j.jacc.2018.08.1037. 30236314 10.1016/j.jacc.2018.08.1037

[13] Torensma B, Hany M, Aarts E, Berends F, van Wagensveld B, Parmar C, et al. Reframing obesity: physiological mechanisms, clinical diagnosis, and implications for metabolic bariatric surgery. Obes Surg. 2025. 10.1007/s11695-025-07870-0. 40369248 10.1007/s11695-025-07870-0

[14] Lemp JM, Nuthanapati MP, Bärnighausen TW, Vollmer S, Geldsetzer P, Jani A. Use of lifestyle interventions in primary care for individuals with newly diagnosed hypertension, hyperlipidaemia or obesity: a retrospective cohort study. J R Soc Med. 2022;115(8):289–99. 10.1177/01410768221077381. 35176215 10.1177/01410768221077381 PMC9340092

[15] Wearing SC, Hennig EM, Byrne NM, Steele JR, Hills AP. Musculoskeletal disorders associated with obesity: a biomechanical perspective. Obes Reviews: Official J Int Association Study Obes. 2006;7(3):239–50. 10.1111/j.1467-789X.2006.00251.x. 10.1111/j.1467-789X.2006.00251.x 16866972

[16] Nobrega S, Champagne N, Abreu M, Goldstein-Gelb M, Montano M, Lopez I, et al. Obesity/overweight and the role of working conditions: a qualitative, participatory investigation. Health Promot Pract. 2016;17(1):127–36. 10.1177/1524839915602439. 26333770 10.1177/1524839915602439 PMC5860907

[17] Lin H-Y, Huang C-K, Tai C-M, Lin H-Y, Kao Y-H, Tsai C-C, Hsuan C-F, Lee S-L, Chi S-C, Yen Y-C. Psychiatric disorders of patients seeking obesity treatment. BMC Psychiatry. 2013;13(1):1. 10.1186/1471-244X-13-1. 23281653 10.1186/1471-244X-13-1 PMC3543713

[18] Dicker D, Sagy YW, Ramot N, Battat E, Greenland P, Arbel R, Lavie G, Reges O. Bariatric metabolic surgery vs Glucagon-Like Peptide-1 receptor agonists and mortality. JAMA Netw Open. 2024;7(6):e2415392–2415392. 10.1001/jamanetworkopen.2024.15392. 38848064 10.1001/jamanetworkopen.2024.15392 PMC11161844

[19] Maan S, Sohail AH, Sulaiman SA, Mansoor L, Cohen EM, Adekolu AA, et al. Metabolic and bariatric surgery versus glucagon-like peptide-1 receptor agonist therapy: a comparison of cardiovascular outcomes in patients with obesity. Am J Surg. 2025;242:116242. 10.1016/j.amjsurg.2025.116242. 39965476 10.1016/j.amjsurg.2025.116242

